# An Improved Adam Optimization Algorithm Combining Adaptive Coefficients and Composite Gradients Based on Randomized Block Coordinate Descent

**DOI:** 10.1155/2023/4765891

**Published:** 2023-01-10

**Authors:** Miaomiao Liu, Dan Yao, Zhigang Liu, Jingfeng Guo, Jing Chen

**Affiliations:** ^1^School of Computer and Information Technology, Northeast Petroleum University, Daqing 163318, China; ^2^Heilongjiang Key Laboratory of Petroleum Big Data and Intelligent Analysis, Daqing 163318, China; ^3^College of Information Science and Engineering, Yanshan University, Qinhuangdao 066004, China

## Abstract

An improved Adam optimization algorithm combining adaptive coefficients and composite gradients based on randomized block coordinate descent is proposed to address issues of the Adam algorithm such as slow convergence, the tendency to miss the global optimal solution, and the ineffectiveness of processing high-dimensional vectors. The adaptive coefficient is used to adjust the gradient deviation value and correct the search direction firstly. Then, the predicted gradient is introduced, and the current gradient and the first-order momentum are combined to form a composite gradient to improve the global optimization ability. Finally, the random block coordinate method is used to determine the gradient update mode, which reduces the computational overhead. Simulation experiments on two standard datasets for classification show that the convergence speed and accuracy of the proposed algorithm are higher than those of the six gradient descent methods, and the CPU and memory utilization are significantly reduced. In addition, based on logging data, the BP neural networks optimized by six algorithms, respectively, are used to predict reservoir porosity. Results show that the proposed method has lower system overhead, higher accuracy, and stronger stability, and the absolute error of more than 86% data is within 0.1%, which further verifies its effectiveness.

## 1. Introduction

The introduction of this study is described in the following sections.

### 1.1. Background

With the rapid development of artificial intelligence, population optimization algorithms [1], the memetic algorithm [2], and first-order optimization methods, such as random gradient descent [3] and gradient descent with momentum (SGDM) [4], have been widely used in the field of machine learning and play an important role in solving optimization problems of complex systems. As a first-order adaptive step stochastic gradient optimizer, the Adam algorithm has gained a lot of attention in the field of numerical optimization for its outstanding computational efficiency and has been widely used in deep learning with impressive results [5]. However, the first-order momentum of the Adam algorithm is an exponentially weighted average of the historical gradients, and the update of the search direction is influenced by the deviation value of the gradient, which leads to slow convergence of the model. While the second-order momentum is accumulated over a fixed time window, and the data do not vary monotonically with the time window. This generates oscillations in the learning rate in the later stages of training and leads to failure of the model convergence. Therefore, it has become a focus of researchers to seek methods to improve the defects of the Adam algorithm in convergence.

### 1.2. Related Work

The Adam algorithm mainly uses momentum and deviation correction methods to achieve stronger search ability. Hence, most of the relevant research studies focus on further improving the performance of the optimizer or combining it with other optimization methods [6]. By assigning a “long-term memory” to the historical gradients, the AMSGrad [7] algorithm is proposed, which solves the convergence problem theoretically. Based on the momentum-accelerated stochastic gradient descent, Ma and Yarats [8] proposed a quasi-hyperbolic weight decay acceleration algorithm and adjusted the hyperparameters. Luo et al. [9] compared the generalization and convergence capabilities of stochastic gradient descent (SGD) and adaptive methods and provided new variants of Adam and AMSGrad, identified as AdamBound and AMSBound, respectively, by using dynamic learning rate variation bounds to achieve an asymptotic and smooth transition from adaptive methods to SGD. Yin et al. [10] proposed a C-Adam algorithm based on the current gradient, predicted gradient, and historical momentum gradient to attain iteratively more accurate search directions by updating the true gradient. Subsequently, a hybrid Adam-based optimization method HyAdamC [11] is proposed, which carefully tunes the search intensity using three-speed control functions: initial, short term, and long term, thus, significantly enhancing the prediction accuracy. Later, some methods were proposed such as AdaGrad [12], Yogi [13], Fromage [14], diffGrad [15], RBC-Adam [16], and TAdam [17].

Although the above optimization algorithms can achieve competent results when used to train neural networks, they still pose the following three problems. First, the algorithms need to determine an optimal search speed at each training step, which may introduce overfitting or affect the training accuracy and testing accuracy [18]. Secondly, the current momentum used in the Adam is prone to inaccurate search directions because of gradient deviations caused by the outliers [17]. Thirdly, such algorithms have difficulty in identifying the current state of the optimized terrain in the solution space spanned by the weights, and therefore, they fail to find the approximate optimal weights.

### 1.3. Contribution

To deal with the above problems, an improved Adam optimization algorithm, combining adaptive coefficients and composite gradients based on randomized block coordinate descent, written ACGB-Adam, is proposed. The contributions and innovations of this article are summarized as follows. (1) To deal with the problem of slow convergence of the Adam algorithm, adaptive coefficients are used for computing the degree of difference between the first-order momentum and the current gradient. This helps to reduce the degree of influence of parameters on the deviated gradient caused by the outlier points, improve the proportion of influence of the parameters on the momentum at the previous moment, avoid the gradient deviation, and enhance the search speed and convergence accuracy. (2) Aiming at the shortcoming that the Adam algorithm tends to miss global optimal solution, the prediction gradient is introduced and combined with the current gradient and the first-order momentum to form a composite gradient, thus, providing a joint determination of the direction of the iterative optimization. This helps to get a more accurate search direction and improve the global search capability, thereby speeding up the search for the global optimal solution. (3) To address the issue of dealing with high-dimensional vectors and the high computational overhead of the Adam algorithm, the randomized block coordinate descent (RBC) is introduced to determine the gradient update mode according to the random variables of the diagonal matrix. This ensures that only one block of the gradient needs to be computed in each iteration instead of the entire gradient. Then, the dynamic balance between the convergence accuracy and the system overhead can be achieved. (4) Combining the above ideas, the ACGB-Adam optimization algorithm is proposed. The optimization performance of the proposed algorithm is verified by standard classification datasets Mnist and CIFAR-10, which is further applied to BP neural networks and compared with optimization methods based on SGD, AdaGrad, Adam, C-Adam, and RBC-Adam. From the experimental results, it can be concluded that the algorithm proposed in this article has better performance, and its convergence speed, stability, and prediction accuracy are higher than those of the other five methods.

## 2. Adam Algorithm

The Adam algorithm is explained in the following sections.

### 2.1. Basic Principles

The Adam algorithm [19] differs significantly from the traditional SGD algorithms. SGD algorithm maintains a single learning rate to update all the weights during training; the AdaGrad algorithm reserves a learning rate for each parameter to improve the performance on sparse gradients; the RMSProp algorithm adaptively reserves a learning rate for each parameter based on the mean of the nearest magnitude of the weight gradient, thereby improving the algorithm's performance on nonstationary problems. Adam algorithm sets independent adaptive learning rates for different parameters by computing the first-order and the second-order momentum estimates of the gradient and gains the advantages of both the AdaGrad and RMSProp algorithms.

Particularly, the Adam algorithm uses not only first-order momentum to maintain the direction of the historical gradient but also second-order momentum to maintain the adaptive state of the learning rate. Besides, it directly considers a sequential setting where samples are displayed sequentially rather than assuming that a large number of training samples are pre-available. Because of these reasons, the Adam algorithm performs well with high computational efficiency and low memory requirements [20]. In recent years, research on the Adam algorithm has flourished, and several variants such as NAdam [21], GAdam [22], AMSGrad [23], Adafactor [24], and Adadelta [25] have been proposed.

### 2.2. Algorithm Flow

In view of accurately describing the Adam algorithm and its improvement, the relevant parameters involved in this article are described in Table 1. The pseudocode of the Adam algorithm is shown in Algorithm 1.

### 2.3. Existing Problems

In deep learning, the Adam algorithm is widely used to solve parameter optimization problems because of its efficient calculation, smaller number of tuning parameters, and high compatibility. However, there are certain shortcomings of this algorithm. Firstly, the model convergence speed is very slow. The first-order momentum in the Adam algorithm is the exponentially weighted average of the historical gradient, which controls the update of the optimization direction. It gets easily affected by the gradient deviation value, leading to poor searchability and slow convergence speed of the model. Secondly, it is easy to miss the global optimal solution. The neural network model often contains a large number of parameters. In a space with extremely high dimensions, the nonconvex objective function often tends to rise and fall, and it is easy to produce the “plateau phenomenon” that causes the training to stop and then miss the global optimal solution.

## 3. ACGB-Adam Algorithm

To solve the problems of the Adam algorithm, the ACGB-Adam algorithm is proposed, which is primarily improved from the following three aspects. (1) To address the slow convergence speed of the Adam algorithm, an adaptive coefficient calculation method is adopted to improve the search direction and reduce the influence of gradient deviation caused by the outliers on the first-order momentum search direction. (2) In view of the issue that the Adam algorithm is easy to miss the global optimal solution, a composite gradient is formed out of the current gradient and the predicted gradient, which enhances the correctness of the search direction, improves the global optimization ability, and further boosts the search efficiency and optimization ability of the algorithm. (3) To reduce the computational cost of the algorithm, the randomized block coordinate descent method is introduced to select variables by modules to calculate the gradient update mode. This contributes to reducing the memory and CPU utilization as much as possible on the premise of ensuring the search performance.

### 3.1. Adjust Gradient Deviation with Adaptive Coefficients

**Figure 1:**
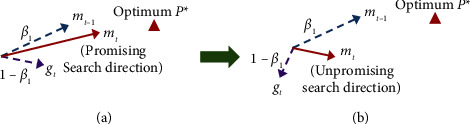
The effect of the desired gradient on the first-order momentum: (a) ordinary gradients and (b) outlier gradients.

In the Adam algorithm, the gradient deviation caused by outliers has a significant impact on the calculation of the first-order momentum. From the exponential weighted average (EWA), it can be noticed that the first-order momentum maintains the movement direction of its historical gradient, so the search direction of the next time is determined by the previous first-order momentum of the current gradient. Subsequently, if the current gradient is far from the global optimal direction, the direction of the first-order momentum will be further away from the approximate optimum, leading to a serious decline in the search ability.  Figure 1 demonstrates the impact of the desired gradient on the first-order momentum. As highlighted in Figure 1(a), the first-order momentum at the current time *m*_*t*_ is calculated by the EWA between the previous momentum *m*_*t*−1_ and the current gradient *g*_*t*_, and the two constant coefficients *β*_1_ and (1 − *β*_1_) are used to obtain the EWA. At this time, the direction of *m*_*t*_ shifts to the direction of *g*_*t*_ if *g*_*t*_ deviates from the desired direction due to the influence of the outliers. Therefore, the search direction at the next time will also be further away from the approximate global optimum *P*^*∗*^, as demonstrated in Figure 1(b).

To improve the slow convergence speed caused by the deviation of the first-order momentum search direction, it is mandatory to confirm whether the current gradient is the deviation gradient caused by the outliers and reduce its impact as much as possible. So, the ACGB-Adam algorithm computes the difference between *m*_*t*−1_ and *g*_*t*_. If this difference is very large, *g*_*t*_ is more likely to affect the search direction at the next moment than the first-order momentum. In this case, the influence of the momentum at the previous time *m*_*t*−1_ will be increased according to their difference degree by an adaptive coefficient to reduce the influence of *g*_*t*_ on *m*_*t*_ as much as possible. The outlier gradient adjustment method based on the adaptive coefficient is expressed as

(1) $$\begin{array}{c} m_{t}=\beta_{1,t}m_{t-1}+\left(1-\beta_{1,t}\right)g_{t}\text{,} \end{array}$$

where *β*_1,*t*_ is the adaptive coefficient, which is proportional to the difference between *m*_*t*−1_ and *g*_*t*_, namely, *β*_1,*t*_ ∝ |*m*_*t*−1_ − *g*_*t*_|. In this article, the method in [17] is used to determine the difference ratio, as mentioned in equation (2). In equation (2), *q*_*t*_ denotes the similarity between *g*_*t*_ and *m*_*t*_ as calculated by equation (3), and *d* represents the vector dimension. *Q*_*t*−1_ is a weighted cumulative sum of *q*_1_,  *q*_2_ ,…,  *q*_*t*−1_, as calculated by equation (4):

(2) $$\begin{array}{c} \beta_{1,t}=\frac{Q_{t-1}}{Q_{t-1}+q_{t}}\text{,} \end{array}$$

(3) $$\begin{array}{c} q_{t}=2d\left(d+\overset{d}{\underset{j=1}{\sum }}\frac{{\left(g_{t,j}-m_{t-1,j}\right)}^{2}}{v_{t-1,j}}\right)\text{,} \end{array}$$

(4) $$\begin{array}{c} Q_{t-1}=\frac{2\beta_{1}-1}{\beta_{1}}Q_{t-2}+q_{t-1}\text{.} \end{array}$$

### 3.2. Combined Predicted Gradient to Form Composite Gradient

**Figure 2:**
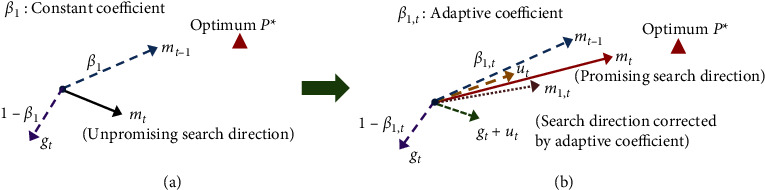
Adjustment of the first-order momentum based on adaptive coefficient and composite gradient: (a) ordinary gradient optimization and (b) the improved gradient optimization.

In the Adam algorithm, the first-order momentum *m*_*t*_ is determined by the current gradient *g*_*t*_ and the historical first-order momentum *m*_*t*−1_. This causes the search direction to be excessively dependent on the historical gradient, making it easy to miss the global optimum. The ACGB-Adam algorithm thus introduces the predicted gradient *u*_*t*_, updates the parameter to be optimized at the next moment by the gradient descent method, and differs it from the historical momentum so that it uses a real gradient update and then merges with the current gradient and the historical first-order momentum to form a composite gradient. This makes it possible to get a more accurate search direction in the next iteration.  Figure 2 illustrates the schematic diagram of the first-order momentum search direction adjustment mechanism integrating adaptive coefficients and composite gradient.

For the first-order momentum before improvement in Figure 2(a), a constant coefficient *β*_1_ is used. Therefore, if *g*_*t*_ moves away from the optimal position *P*^*∗*^ in a direction deviated from the desired direction, *m*_*t*_ will continue its movement in the direction of *g*_*t*_. In Figure 2(b), *m*_1,*t*_ is the search direction corrected by the adaptive coefficient *β*_1,*t*_. As compared to the direction of *m*_*t*_ in Figure 2(a), *m*_1,*t*_ will approach the global optimal position in a more accurate direction. Therefore, to adjust the gradient effect of outliers, an adaptive coefficient is introduced. Because of this, the influence of the outliers of the first-order momentum at the previous moment is as small as possible while calculating the current first-order momentum. Thus, a more potential search direction can be effectively determined, and the search for the global optimal solution can be accelerated. Secondly, based on the use of an adaptive coefficient to correct the search direction, the predicted gradient *u*_*t*_ is introduced, and the search direction *m*_*t*_ is formed together with the current gradient *g*_*t*_ and the historical first-order momentum *m*_*t*−1_. It can be observed that, by introducing the predicted gradient, on the basis of the adjustment of *m*_1,*t*_, the search direction formed can be further closer to *P*^*∗*^ to avoid missing the global optimal solution. Therefore, the convergence accuracy of the algorithm is improved.

### 3.3. Gradient Update Mode Based on Randomized Block Coordinate Descent

As a simple and effective method, SGD is often used to learn linear classifiers. However, when dealing with high-dimensional vector data, the full gradient descent mode in SGD is not easy to be implemented in parallel. Therefore, this article introduces the random block coordinate method to optimize the Adam algorithm, which can not only handle high-dimensional vectors but also can avoid calculating the complete gradient of all dimensional data in each iteration, thus saving the computing cost and reducing the system overhead on the premise of ensuring the convergence speed and optimization accuracy.

#### 3.3.1. RBC Algorithm

RBC is a random optimization algorithm [26]. In each iteration, a coordinate (block) is randomly selected, and its variables are updated in the coordinate gradient direction. If *f* is a convex smooth function and its gradient *L*_*i*_ (*iϵ*{1,2, ..., *N*}) is a Lipschitz continuous number, the flow of the RBC algorithm is as follows: wherein, *x*_*t*_ denotes the parameter vector to be updated. The RBC algorithm is as shown in Algorithm 2.

RBC algorithm has been widely used to address large-scale optimization problems because of its low computation and update cost [16] and its good optimization effect. For instance, Hu and Kwok [27] studied the learning of scalable nonparametric low-rank kernels, and Zhao et al. [28] proposed an accelerated small-batch random block optimization algorithm. Moreover, several machine learning algorithms can be optimized with the help of RBC. For instance, Singh et al. [29] improved the gradient projection algorithm by using RBC, and Xie et al. [30] combined the RBC algorithm with mean-variance optimization.

#### 3.3.2. Gradient Calculation Based on RBC

In this article, a new gradient calculation method is proposed based on the RBC method. Let *D*_*t*_(*t*=0,1,2, ..., *N*) be a *n*-dimensional diagonal matrix in the *t*th iteration, and the *i*th element on the diagonal is denoted as *d*_*i*_^*t*^. Here, *d*_*i*_^*t*^ is a Bernoulli random variable that satisfies the independent identically distributed, i.e., *d*_*i*_^*t*^ ∈ {0,1}, 1 ≤ *i* ≤ *N*:

(5) $$\begin{array}{c} D_{t}=\left[\begin{array}{cc} 1 & \begin{array}{cc} 0 & \begin{array}{cc} \cdots & 0 \end{array} \end{array}\\ \begin{array}{c} 1\\ \begin{array}{c} \vdots \\ 0 \end{array} \end{array} & \begin{array}{cc} \begin{array}{c} 0\\ \begin{array}{c} \vdots \\ \cdots \end{array} \end{array} & \begin{array}{c} \begin{array}{cc} \cdots & \vdots \end{array}\\ \begin{array}{c} \begin{array}{cc} \ddots & \vdots \end{array}\\ \begin{array}{cc} \cdots & 1 \end{array} \end{array} \end{array} \end{array} \end{array}\right]\text{.} \end{array}$$

**Figure 3:**
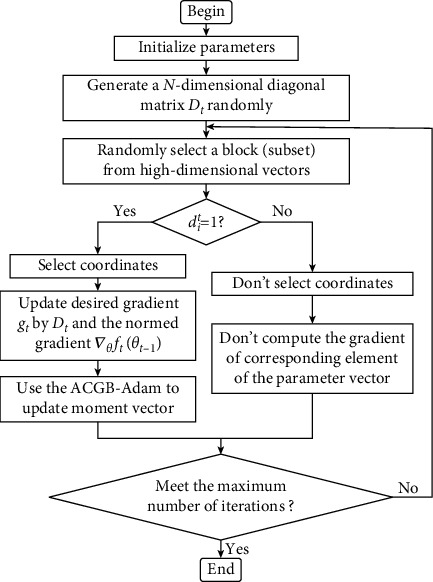
Flowchart of the gradient calculation based on RBC.

The RBC method is used to randomly select a block (subset) from the whole element of a high-dimensional vector through equation (5) If *d*_*i*_^*t*^=1, which means that the corresponding coordinates are selected, then the ACGB-Adam algorithm is executed for gradient calculation; if *d*_*i*_^*t*^=0, which means that the corresponding coordinates are not selected, then the gradient update calculation is not performed. Thus, in each round of gradient updating, only one block (subset) of the gradient has to be computed, and the first-order and second-order momentum are calculated based on this. Moreover, it is not necessary to calculate the entire gradient. Therefore, compared with the other full gradient descent algorithms, the optimization method based on randomized block coordinate descent may save a lot of computing costs and reduce CPU utilization as well as memory utilization while ensuring the convergence of the algorithm. The specific calculation process is shown in Figure 3.

### 3.4. ACGB-Adam Algorithm Process

The ACGB-Adam algorithm process is described in the following sections.

#### 3.4.1. Overall Architecture of Algorithm

**Figure 4:**
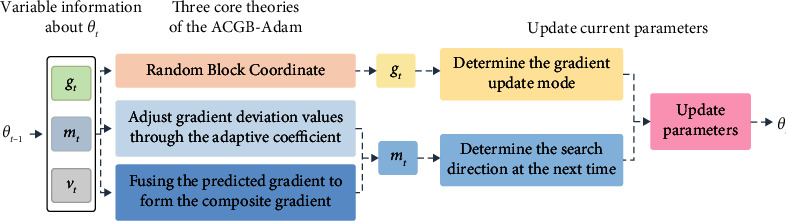
The architecture of ACGB-Adam.

The overall architecture of the ACGB-Adam algorithm is shown in Figure 4, which mainly includes three core modules: the random block coordinate method, the adjustment of gradient deviation values through adaptive parameters, and the composite gradient.

The general strategy of the ACGB-Adam algorithm is to integrate the above three modules and apply three optimization methods to solve problems in parameter updating so as to improve the convergence speed, global optimization ability and reduce the system overhead. First, the current gradient update mode is optimized by RBC, which can avoid calculating all gradients and reduce the system overhead. Secondly, through the adaptive parameters, the algorithm could calculate the coefficient proportion of the first momentum adaptively according to the difference between the current gradient and the first momentum at the last time so as to minimize the influence of the outlier gradient and optimize the search direction and search speed. Finally, the composite gradient combines the predicted gradient, the current gradient, and the first momentum of the last time to form the final search direction, aiming to further approach the global optimal position and improve the global search ability of the algorithm.

#### 3.4.2. ACGB-Adam Algorithm Process

The overall algorithm flow of ACGB-Adam is shown in Algorithm 3.

## 4. Experiment and Analysis

The experiment and analysis are described in the following sections.

### 4.1. Standard Datasets and Experimental Setup

**Figure 5:**
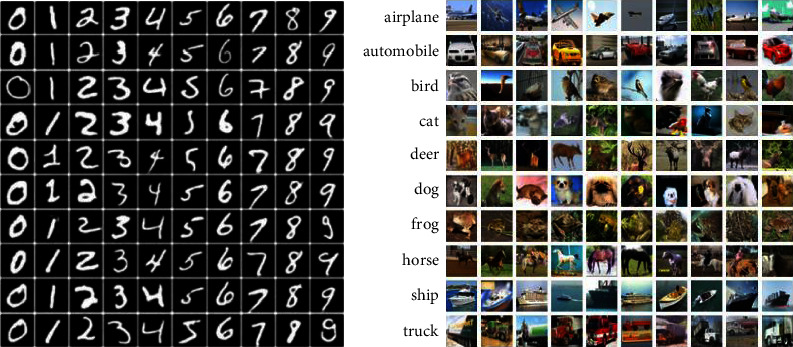
Samples of standard datasets: (a) Mnist dataset handwritten instances and (b) CIFAR-10 dataset picture category instances.

To evaluate the performance of the ACGB-Adam algorithm, experiments were carried out on two standard datasets (Table 2) used for classification. The proposed algorithm was further compared with the stochastic gradient descent (SGD), the adaptive gradient (AdaGrad), the adaptive moment estimate (Adam), the Adam optimization algorithm based on adaptive coefficients (A-Adam), Adam optimization algorithm based on composite gradient (C-Adam), and Adam optimization algorithm based on randomized block coordinate descent (RBC-Adam) algorithms (Figure 5(a)).

1. *Mnist Dataset*. The Mnist dataset [31] developed by the US postal system is a classic dataset for image recognition. In this dataset, 70000 digital pictures of 0∼9 handwritten by 250 different people are counted. These numbers have been standardized in size and are located in the center of the image. Some examples of handwriting in the dataset are represented in Figure 5(a).
2. *CIFAR-10 Dataset*. The CIFAR-10 dataset [32] is used for identifying universal objects which consists of 60000 RGB images. Compared with the handwritten characters, this dataset contains pictures of real objects in the real world. The noise is large, and the proportions and characteristics of objects are different, which lead to great difficulties in recognition.  Figure 5(b) lists ten classes in the dataset, and each class shows ten pictures randomly.
3. *Experimental Setting.* MATLAB is used for the simulation of experiments. The operating system is Win10, the CPU is Intel i7–1065G7, the primary frequency is 1.30 GHz, the memory is 16 GB, and the SSD capacity is 512 GB. To improve the comparability of the results, the six comparison algorithms involved in the experiment all use the same parameter settings. The main superparameters are as follows: *α* = 0.001, *β*_1_ = *β*_2_ = 0.9, and the maximum number of iterations is 100. MSE and accuracy are used as performance evaluation indicators of algorithm training and classification accuracy.

### 4.2. Experimental Results of the Standard Dataset

The experimental results of the standard dataset are explained in the following sections.

#### 4.2.1. Mnist Experimental Results

**Figure 6:**
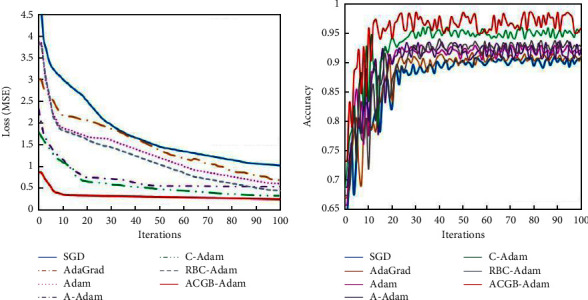
Experimental results of the Mnist dataset: (a) MSE and (b) accuracy.

Figure 6 represents the training error loss and classification accuracy of the six algorithms on the Mnist. The training error and test accuracy at the 100th iteration are shown in Table 3. It can be observed from Figure 6 and Table 3 that, as the number of iterations increases, each algorithm gradually converges on the training set, and the classification accuracy on the test set keeps improving. Compared with the other six algorithms, the ACGB-Adam algorithm quickly converges to the stable state, has the smallest error loss value, and has the highest classification accuracy of 0.959. This indicates that the algorithm proposed in this article has a good classification effect.

#### 4.2.2. CIFAR-10 Experimental Results

**Figure 7:**
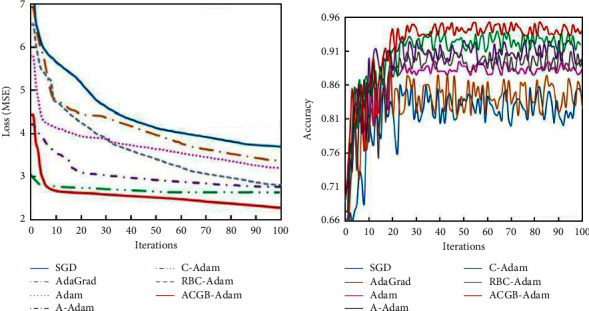
Experimental results of the CIFAR-10 dataset: (a) MSE and (b) accuracy.

Figure 7 demonstrates the training error of algorithms on the CIFAR-10 dataset, along with the classification accuracy of the test set. The training error and test accuracy at the 100th iteration are shown in Table 4. It can be observed from Figure 7 and Table 4 that the training error of the ACGB-Adam algorithm in the early stage of iterations reduces quickly and gradually tends to be stable. With the increase in iterations, the error loss of the algorithm still decreases steadily. Compared with the other six algorithms, the ACGB-Adam algorithm has the smallest error loss value and the highest classification accuracy of 0.941. From the experimental results on the CIFAR-10 dataset, it can be inferred that the proposed algorithm in this article has better optimization performance than the other six algorithms in terms of convergence speed, accuracy, stability, and classification accuracy.

#### 4.2.3. Memory and CPU Usage Rate Analysis

**Figure 8:**
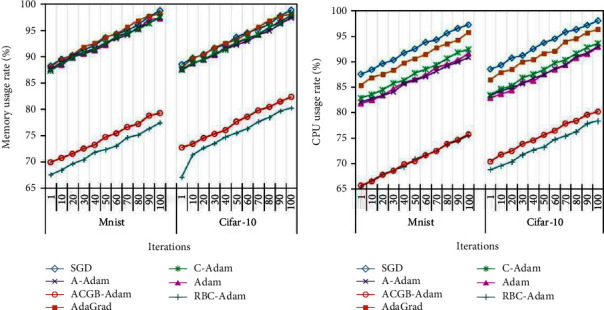
Change of (a) memory usage rate and (b) CPU usage rate with iterations.

For the two standard datasets, the changes in memory and CPU utilization of the seven algorithms with the number of iterations are illustrated in Figure 8 and Table 5.

It can be observed from Table 5 and Figure 8 that, with the increase of iteration times, the memory and CPU utilization of each algorithm increase gradually. Under the same conditions, the memory and CPU utilization of the RBC-Adam algorithm is the lowest, followed by the ACGB-Adam algorithm proposed in this article. The difference between the memory and CPU utilization rates of the two algorithms is less than 2%. The specific experimental results are shown in Table 6. It can be seen from Tables 5 and 6 that although the computing cost of the RBC-Adam algorithm is slightly lower than the ACGB-Adam algorithm, its training error and classification accuracy are far lower than those of the proposed algorithm. Altogether, the ACGB-Adam algorithm proposed in this article can achieve a dynamic balance in convergence and computing cost. On the premise of improving the convergence speed and accuracy, it can reduce the memory and CPU utilization to the greatest extent and has good comprehensive optimization performance.

### 4.3. Reservoir Porosity Prediction

To further verify the effectiveness and utility of the algorithm proposed, the reservoir porosity in the real work area was predicted by a BP neural network based on the ACGB-Adam algorithm.

#### 4.3.1. Data Preparation and Preprocessing

**Figure 9:**
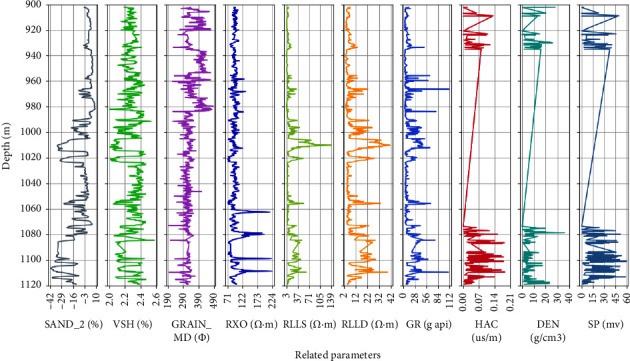
Distribution of sample data in the work.

**Figure 10:**
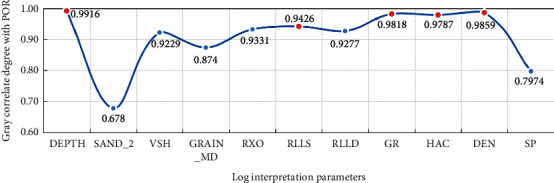
Gray correlation degree of the relevant parameters.

As shown in Figure 9, the sample data are from the real data of two wells, *A* and *B*, in an exploration area. The logging depth is 900∼1120 m, including 1492 records and 11 logging parameters. To achieve efficient and accurate porosity prediction, the grey correlation analysis method [33] is used to select parameters with high correlation with porosity as input parameters of the neural network, namely, Depth, RLLS (shallow investigate double lateral resistivity log), GR (natural gamma ray), HAC (high-resolution interval transit time), and DEN (density), as represented in Figure 10. This helps to improve the data processing efficiency on the premise of ensuring prediction accuracy.

It can be assumed that these five parameters that have a significant impact on porosity are different in nature and usually have distinct dimensions and orders of magnitude. In case the level difference between the parameters is too large, the influence of the parameters with higher values will be highlighted, and the effect of the parameters with lower values will be weakened. To ensure the comparability of the data, this article uses the deviation normalization method [33] to preprocess the data and eliminates the influence of the dimension and the value of the variable itself on the results.

#### 4.3.2. Model Performance Analysis

**Figure 11:**
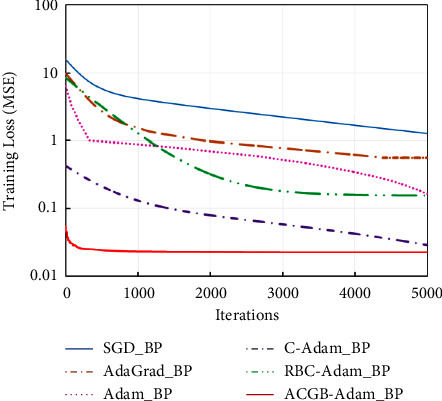
The iterative curves of the methods.

The preprocessed data were taken as sample data, and the training set and test set were divided in the ratio of 8 : 2. The BPNN model is set as follows: the number of hidden layers was 1, including 5 neurons, the transfer function was Tansig, the learning rate was 0.001, and the maximum number of iterations was 5000. Using MSE and RMSE as the model performance evaluation indices, the proposed ACGB-Adam_BP model was compared with five methods, namely, SGD_BP, AdaGrad_BP, Adam_BP, C-Adam_BP, and RBC-Adam_BP. The final training error and test error of various methods are enlisted in Table 7, in which the minimum values of MSE and RMSE are shown in bold, and the iterative error curve is shown in Figure 11.

It can be seen from Table 7 and Figure 11 that the BPNN based on the ACGB-Adam algorithm generates the lowest error in the training set and the test set and tends to be stable as soon as possible. The convergence speed is much better than the other five comparison algorithms. This indicates that the proposed algorithm has better optimization performance.

#### 4.3.3. Porosity Prediction Results

**Figure 12:**
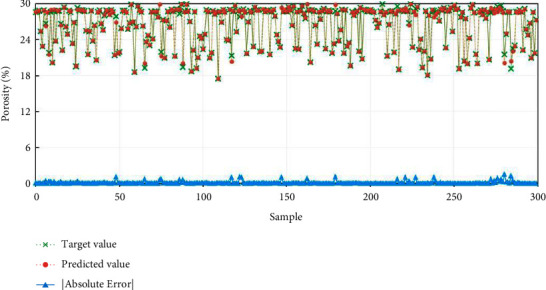
Porosity prediction results based on the ACGB-Adam_BP method.

To further observe the above results intuitively and validate the effectiveness and correctness of the method proposed in this article for porosity prediction, the prediction results of the BP model based on the ACGB-Adam optimization algorithm are visually analyzed in terms of 300 test samples, as highlighted in Figure 12. Due to space constraints, the error analysis results on the training set are not shown in the article. From the comparison curve between the predicted value and the actual value of porosity, it can be observed that the BP neural network model based on the ACGB-Adam optimization algorithm has a relatively ideal prediction result, and the predicted abnormal value of porosity is quite less. The absolute error of more than 86% of the data is within 0.1%, which signifies the high prediction accuracy of the proposed algorithm.

## 5. Conclusion

Starting with the improvement of the Adam algorithm to heighten the convergence speed, accelerating the search for the global optimal solution, and enhancing the high-dimensional data processing ability, the Adam optimization algorithm combining adaptive coefficients and composite gradients based on randomized block coordinate descent is proposed, which enhances the performance of the algorithm. Through theoretical analysis and numerical experiments, the following conclusions can be drawn:

1. The gradient deviation caused by the outliers is crucial to the convergence speed and solution precision of the Adam algorithm. Using an adaptive coefficient to adjust the difference between the first-order momentum and the current gradient can help in reducing the influence of parameter proportion of deviation gradient, improving the slow convergence speed of the Adam algorithm, boosting the search speed, and improving the convergence accuracy.
2. By introducing the prediction gradient and combining the current gradient and the first-order momentum to form a composite gradient, an accurate search direction can be obtained in the subsequent iteration, and then, the global optimization ability of the algorithm could be enhanced.
3. In the process of gradient updating, the RBC method is used to determine the gradient calculation method by randomly selecting variables from the parameter subset. This can reduce the calculation cost as much as possible on the premise of ensuring the convergence of the algorithm, enhance the processing ability of the algorithm for high-dimensional data, and maintain a good balance between the optimization accuracy and the system overhead.
4. The test results on Mnist and CIFAR-10 standard datasets for classification indicate that the ACGB-Adam algorithm is significantly superior to SGD, AdaGrad, Adam, *A*-Adam, *C*-Adam, and RBC-Adam algorithms in terms of convergence speed and optimization accuracy. Although the proposed method is slightly higher than the RBC-Adam algorithm in terms of memory and CPU utilization, it can achieve a decent balance between convergence and system overhead. According to the evaluation indices, the proposed algorithm has better performance advantages compared with the other five algorithms, which validates the effectiveness of the algorithm improvement.
5. The BPNN model based on the ACGB-Adam algorithm is applied to reservoir porosity prediction. The experimental results suggest that, as compared to the BPNN model based on Adam and its variants, the maximum reduction of MSE and RMSE of the proposed model in this article is approximately 86.30% and 62.99%, respectively, which achieves higher accuracy in porosity prediction, verifies the superiority of the proposed algorithm, and extends the application field of the algorithm.

The method proposed in this article enhances the performance of the Adam optimization algorithm to a certain extent, but does not consider the impact of the second-order momentum and different learning rates on the performance of the original algorithm. Therefore, the follow-up research can focus on the optimization and improvement of the second-order momentum and learning rate and conduct in-depth and detailed research on the parts not involved in this algorithm. This can help to attain better optimization performance.

## Figures and Tables

**Algorithm 1:**
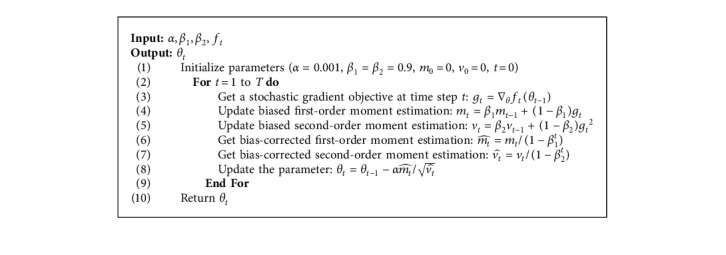
: Adam.

**Algorithm 2:**
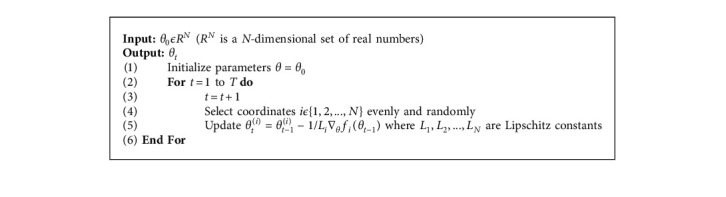
: Randomized block coordinate descent (RBC).

**Algorithm 3:**
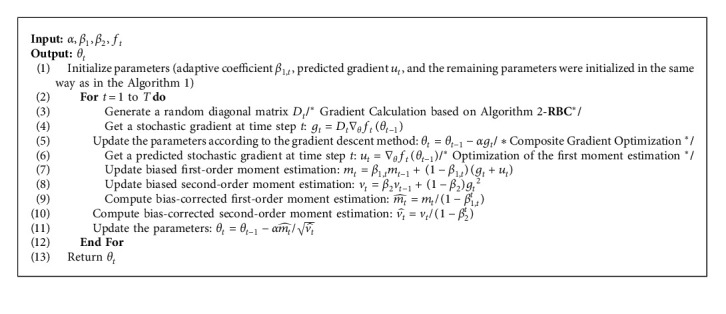
: ACGB-Adam.

**Table 1:** Description of parameters of Adam algorithm and its improvement.

Parameters	Description
*α*	Learning rate
*β*_1_, *β*_2_	Exponential decay rate of the first-order and second-order moment estimation, respectively
*T, t*	The maximum iterations and the current *t* time step, respectively
*β*_1_^*t*^, *β*_2_^*t*^	Product of exponential decay rate of the first and second-order moment estimation at *t* time step, respectively, (1 − *β*_1_)∑_*i*=1_^*t*^*β*_1_^*t*−*i*^=1 − *β*_1_^*t*^ and (1 − *β*_2_)∑_*i*=1_^*t*^*β*_2_^*t*−*i*^=1 − *β*_2_^*t*^
*m*_*t*_	The first-order moment vector at *t* time step
*v*_*t*_	The second-order moment vector at *t* time step
*g*_*t*_	Current gradient at *t* time step
*β*_1,*t*_	Adaptive coefficient
*u*_*t*_	Prediction gradient
*D*_*t*_	Random diagonal matrix at *t* time step
*d*_*i*_^*t*^	The *i*th diagonal element of *D*_*t*_ with independent identical Bernoulli distribution
*θ*_*t*_	The parameter that needs to be optimized
*f*_*t*_	The sequence of the smooth convex loss function
*P* ^*∗*^	Global optimal position

**Table 2:** Basic information of the Mnist and CIFAR-10 datasets.

Datasets	Classes	Image type	Features	Number of training data	Number of test data
Mnist	10	Grayscale	784	60000	10000
CIFAR-10	10	RGB	3072	50000	10000

**Table 3:** MSE and accuracy on Mnist dataset.

Methods	SGD	AdaGrad	Adam	*A*-Adam	*C*-Adam	RBC-Adam	ACGB-Adam
MSE	1.021	0.675	0.607	0.532	0.325	0.449	0.253
Accuracy	0.908	0.913	0.918	0.926	0.952	0.926	0.959

**Table 4:** MSE and accuracy on the CIFAR-10 dataset.

Methods	SGD	AdaGrad	Adam	*A*-Adam	*C*-Adam	RBC-Adam	ACGB-Adam
MSE	3.690	3.364	3.191	2.757	2.635	2.798	2.287
Accuracy	0.837	0.826	0.885	0.90	0.92	0.901	0.941

**Table 5:** Comparison of the memory and CPU usage rate (%).

	Dataset	SGD	AdaGrad	Adam	*A*-Adam	*C*-Adam	RBC-Adam	ACGB-Adam
Memory usage rate	Mnist	98.7	98.1	97.2	97.4	97.5	77.4	79.2
	CIFAR-10	98.8	98.0	97.7	97.3	97.9	80.2	82.3

CPU usage rate	Mnist	97.2	95.8	91.6	90.8	92.4	75.5	75.7
	CIFAR-10	98.0	96.3	92.9	93.0	93.6	78.3	80.1

**Table 6:** Performance comparison of RBC-Adam and ACGB-Adam.

	Dataset	MSE	Accuracy	Memory usage rate	CPU usage rate
RBC-Adam	Mnist	0.449	0.926	77.4	75.5
	CIFAR-10	2.798	0.901	79.2	82.3

ACGB-Adam	Mnist	0.253	0.959	80.2	78.3
	CIFAR-10	2.287	0.941	82.3	80.1

**Table 7:** Training error and test error of six methods.

	Training error					
	SGD_BP	AdaGrad_BP	Adam_BP	*C*-Adam_BP	RBC-Adam_BP	ACGB-Adam_BP
MSE	1.174293	0.51372	0.095824	0.021837	0.101693	**0.019725**
RMSE	1.083648	0.716743	0.309555	0.147773	0.318893	**0.140446**

Test error						
MSE	SGD_BP	AdaGrad_BP	Adam_BP	*C*-Adam_BP	RBC-Adam_BP	ACGB-Adam_BP
	1.267104	0.566655	0.163862	0.028447	0.155603	**0.022442**
RMSE	1.125657	0.752772	0.404799	0.168662	0.394465	**0.149807**

The minimum values of MSE and RMSE in Table 7 is shown in bold.

## Data Availability

No data were used to support the findings of the study.
